# Otology/Neurotology recommendations – Choosing Wisely campaign

**DOI:** 10.1186/s40463-019-0381-4

**Published:** 2019-11-08

**Authors:** Andrew K. Ma, Julian Nedzelski, Joseph Chen, Trung Le, Paul Mick, Jane Lea, David Morris, Margaret Aron, Sumit Agrawal, Lorne Parnes, Tamara Mijovic, Vincent Lin, Otology/Neurotology subspecialty group of the Canadian Society of Otolaryngology – Head & Neck Surgery

**Affiliations:** 1 Department of Otolaryngology – Head and Neck Surgery, University of Toronto, Toronto, Canada; 2 Department of Otolaryngology – Head and Neck Surgery, Sunnybrook Health Sciences Centre, 2075 Bayview Avenue, M-Wing, M1-102, Toronto, ON M4N 3M5 Canada; 3 Division of Otolaryngology, University of British Columbia, Vancouver, Canada; 4 Division of Otolaryngology, Dalhousie University, Halifax, Canada; 5 Division of Oto-Rhino-Laryngology and Head & Neck Surgery, Université de Sherbrooke, Sherbrooke, Canada; 6 Department of Otolaryngology – Head and Neck Surgery, Western University, London, Canada; 7 Department of Otolaryngology – Head and Neck Surgery, McGill University, Montreal, Canada

**Keywords:** Otology/neurotology, Choosing wisely Canada, Best practice

## Abstract

The Choosing Wisely Canada Campaign aims to raise awareness amongst physicians and patients regarding unnecessary tests and treatment. The otology/neurotology subspecialty group within the Canadian Society of Otolaryngology – Head & Neck Society developed a list of five common otologic presentations to help physicians deliver high quality effective care: (1) Don’t order specialized audiometric and vestibular testing to screen for peripheral vestibular disease, (2) Don’t perform computed tomography or blood work in the evaluation of sudden sensorineural hearing loss, (3) Don’t perform auditory brain responses (ABR) in patients with asymmetrical hearing loss, (4) Don’t prescribe oral antibiotics as first line treatment for patients with painless otorrhea associated with tympanic membrane perforation or tympanostomy tube, and (5) Don’t perform particle repositioning maneuvers without a clinical diagnosis of posterior canal benign paroxysmal positional vertigo.

## Introduction

Choosing Wisely Canada is a campaign aiming to raise awareness amongst physicians and patients regarding unnecessary tests and treatments, and to encourage smart and effective healthcare decision making. In an increasingly resource-conscious healthcare environment, it is prudent to constantly reassess and optimize the efficiency of provision of healthcare. Multiple physician specialty groups have contributed to the development of Choosing Wisely Canada. The goal is to identify and evaluate common clinical scenarios, with associated commonly ordered tests and investigations, many of which may offer no diagnostic or therapeutic benefit, or even cause undue harm to the patient.

The Canadian Society of Otolaryngology – Head and Neck Surgery (CSOHNS) is a proud partner of the Choosing Wisely Canada campaign. Representing practicing as well as in-training otolaryngologists, the CSOHNS aims to improve patient care via education, promotion of research, dissemination of information, scientific advancement, and maintenance of high professional and ethical standards.

Otologic complaints represent some of the most common referrals to general otolaryngologists and family physicians alike. The Otology/Neurotology recommendations therefore aim to offer best practice approaches to help the physician and patient navigate common otologic presentations.

## Methods

This list of recommendations was created by the Otology & Neurotology subspecialty group of the Canadian Society of Otolaryngology – Head & Neck Surgery. Members of the group, representing the national leaders within the subspecialty were asked to create a list of recommendations for unnecessary tests that were seen to be commonly ordered or unnecessary interventions that were commonly performed. These unnecessary tests and interventions can be invasive and incur risk to patients, in addition to unwarranted costs to our public healthcare system. The evidence was then reviewed to further refine the recommendations. The final version of the list was then circulated and approved by the members of the group. This work has been submitted as a letter to the editor as it represents expert opinion based on best available literature.

## Results

### Don’t order specialized audiometric and vestibular neurodiagnostic tests in an attempt to screen for peripheral vestibular disease [1, 2]

The diagnosis of the dizzy patient should be guided by the presenting symptoms and office examination. Tests such as ABR (auditory brainstem response), ECOG (electrocochleography), ENG/VNG (electronystagmography/ videonystagmography), VEMP (vestibular evoked myogenic potential), vHIT (video head impulse test), CDP (computerized dynamic posturography) and RCT (rotational chair testing) should only be ordered if clinically indicated. In general, advanced balance tests should be ordered and interpreted by otolaryngologists with specialized training in the diagnosis and treatment of vestibular disorders (otologists/neurotologists). Clinical indications for testing can include: side localization and stage of progression for Meniere’s disease, assessment of central compensation for acute vestibular loss and confirmation of superior semicircular canal dehiscence syndrome. Specialized tests are rarely indicated in the management of benign paroxysmal positional vertigo.

### Don’t perform computed tomography or blood work in the evaluation of a patient with sudden sensorineural hearing loss (SSNHL) given its presumed viral etiology [3]

According to the American Academy of Otolaryngology - Head and Neck Surgery, sudden sensorineural hearing loss (SSNHL) is defined as a rapid-onset subjective sensation of hearing impairment occurring over a 72-h period. The audiometric criterion required for the diagnosis of SSNHL is the loss of at least 30 dB affecting three consecutive frequencies, without an identifiable underlying condition after thorough history and physical examination.

Blood work which typically would consist of a CBC, differential and electrolytes along with an autoimmune panel are often normal and would not change initial clinical management if abnormal. The CT scan which is done to rule out central causes is not sensitive enough to pick up most cases of retrocochlear pathology. MRI scans should be considered instead. If verified to be sensorineural with audiometric testing, urgent treatment with steroid therapy can be initiated. There is no role for antiviral treatment, thrombolytics or vasoactive substances.

### Don’t perform auditory brainstem responses (ABR) in patients with asymmetrical hearing loss. Asymmetrical hearing loss is defined as bone conduction threshold difference of: (a) 20 dB threshold difference at a single frequency, (b) 15 dB threshold difference at 2 frequencies, (c) 10 dB threshold difference at 3 frequencies [4–6]

If there is no obvious cause of the asymmetry such as unilateral trauma or unilateral noise exposure like gun blasts, a MRI should be ordered. MRI scans are superior in sensitivity for detecting retrocochlear pathologies such as vestibular schwannoma when compared to ABR testing.

### Don’t use oral antibiotics as a first line treatment for patients with painless ear drainage associated with a tympanic membrane perforation or tympanostomy tube unless there is evidence of developing cellulitis in the external ear canal skin and pinna [7–10]

First line therapy constitutes a short course of topical antibiotic/steroid drops. The potential ototoxicity of any topical medication entering the middle ear space should be considered in selecting an appropriate agent. Where available, fluoroquinolone combination preparations (e.g., ciprofloxacin and dexamethasone) should be used as a first choice and caution should be exercised in using topical aminoglycosides. Microdebridement and further assessment should be considered in the following circumstances: (a) failure to respond after a 7 day course, or (b) where follow up does not permit a clear view of a normal tympanic membrane allowing the exclusion of more sinister middle ear disease such as cholesteatoma.

### Don’t perform particle repositioning maneuvers (Epley or Semont) without a clinical diagnosis of posterior semicircular canal benign paroxysmal positional vertigo in the affected ear [11]

Posterior semicircular canal benign paroxysmal positional vertigo (BPPV) should be diagnosed and confirmed with a positive Dix-Hallpike test, and only then should a particle repositioning maneuver be performed. If a patient with positional vertigo has a Dix-Hallpike test that is repeatedly negative or results in atypical nystagmus, less common BPPV variants or central positional vertigo should be considered.

## Discussion

These statements represent best practice recommendations based on expert opinion and a thorough vetting of the available literature. However, it is important to understand that these recommendations serve as a guideline for discussion between physicians and patients, and should not be interpreted as “rules”. Furthermore, these recommendations are not meant to be used to establish coverage decisions or payments by insurers. The physician must determine carefully evaluate each specific clinical encounter in order to provide the best management for the patient.

## Data Availability

Data sharing is not applicable to this article as no datasets were generated or analysed during the current study.
